# Vestibular migraine or Meniere's disease: a diagnostic dilemma

**DOI:** 10.1007/s00415-022-11532-x

**Published:** 2022-12-23

**Authors:** Jing-Yu Chen, Zhao-Qi Guo, Jun Wang, Dan Liu, E. Tian, Jia-qi Guo, Wei-Jia Kong, Su-Lin Zhang

**Affiliations:** 1 Department of Otorhinolaryngology, Tongji Medical College, Union Hospital, Huazhong University of Science and Technology, No. 1277 Jiefang Avenue, Wuhan City, 430022 Hubei Province People’s Republic of China; 2 Institute of Otorhinolaryngology, Tongji Medical College, Union Hospital, Huazhong University of Science and Technology, Wuhan, China

**Keywords:** Meniere’s disease, Vestibular migraine, Dizziness, Vertigo, Audiometry, Vestibular function tests

## Abstract

Meniere’s disease (MD) represents one of the vertigo disorders characterized by triad symptoms (recurrent vertigo, fluctuating hearing loss, tinnitus or ear fullness). The diagnosis of MD relies on the accurate and detailed taking of medical history, and the differentiation between MD and vestibular migraine (VM) is of critical importance from the perspective of the treatment efficacy. VM is a highly prevalent vertigo condition and its typical symptoms (headache, vestibular symptoms, cochlear symptoms) mimic those of MD. Furthermore, the misdiagnosis in MD and VM could lead to VM patients mistakenly receiving the traumatic treatment protocol designed for MD, and sustaining unnecessary damage to the inner ear. Fortunately, thanks to the advances in examination technologies, the barriers to their differentiation are being gradually removed. These advances enhance the diagnostic accuracy of vertigo diseases, especially VM and MD. This review focused on the differentiation of VM and MD, with an attempt to synthesize existing data on the relevant battery of differentiation diagnosis (covering core symptoms, auxiliary tests [audiometry, vestibular tests, endolymphatic hydrops tests]) and longitudinal follow-up. Since the two illnesses are overlapped in all aspects, no single test is sufficiently specific on its own, however, patterns containing all or at least some features boost specificity.

## Introduction

### Meniere’s disease (MD) and vestibular migraine (VM): a short overview.

MD accounted for a substantial part of peripheral vestibular diseases, with about 10 ~ 13% of peripheral vertigo ending up being diagnosed as MD [1, 2]. MD can be recurrent vertigo, fluctuating and progressing sensorineural hearing loss, aural symptoms, such as tinnitus and ear stuffy [3]. Reported prevalence of MD ranging from a marginal rate of 3.5 per 100,000 and to a staggering 513 per 100,000 [4–7]^.^ This disparity might be ascribed to methodological differences or the difficulty in distinguishing between MD and other diseases, especially VM.

VM represents one of the most frequent culprit for recrudescent vertigo [8]. As the most frequent etiology of dizziness in the United States, the 1-year prevalence of VM estimated at 2.7% of the overall population, higher than the 1-year prevalence of BPPV (1.6%) and MD (0.2~0.5%) [9]. VM is a disorder with a wide array of clinical presentations, including migraine with or without aura and vestibular symptoms. As an emerging disease entity, VM is still underdiagnosed by other clinicians as compared with headache specialists. VM patients tend to be misdiagnosed as MD by otorhinolaryngologists, even though VM victims were 5~10 times more than their MD counterparts in the general population [10].

### Similarities and problems

Multiple studies have demonstrated a remarkable overlap between VM and MD. Of note, migraine occurs more often in patients with MD relative to the general population [11]. When compared to age- and sex-matched controls, Radtke et al*.* discovered that individuals with MD had a twice-as-high lifetime prevalence of migraine[12]. Additionally, VM patients may also experience tinnitus, auditory fullness, and changeable sensorineural hearing loss [12–14]. The overlap between VM and MD is considerable, suggesting that more caution should be exercised in differentiating the two conditions and establishing the final diagnosis. Therefore, an improved diagnostic battery may be of great help.

Despite the high reliability and validity of the criteria [3, 15], the borderline between vertigo and headache remains thin, and the differentiation between MD and VM remains a huge challenge. Mounting incomplete diagnoses are made especially when vestibular symptoms and headaches appear at different time. Vuralli et al. reported that 51.5% of VM patients had been previously misdiagnosed as having MD [16]. Moreover, comorbidity is a frequent cause of diagnostic uncertainty. Neff et al. demonstrated that patients with Ménière’s disease plus Vestibular Migraine (MDVM) amounted to about 25% of their subjects [17]. The concomitant existence of the two conditions making the differentiation between MD and VM extremely difficult [18]. The differentiation between MD and VM is a genuine diagnostic dilemma, and, as a consequence, ambiguous diagnosis or delayed treatment may affect patients’ quality of life and pose financial burdens on both victims and society at large. Although some studies examined the benefits of migraine treatments in MD patients [19], which are likely to improve their quality of life. Nonetheless, with VM patients, inappropriate treatments, such as traumatic surgery, can cause unnecessary impairment to inner ear functions.

To address this problem, this review explored the optimal methods of differential diagnosis between MD and VM on the basis of existing studies, trying to find approaches to diagnose the two conditions with higher accuracy. As with any other diseases, differentiating MD and VM based on an objective diagnostic battery rather than solely on the subjective symptoms of the patients can help researchers and clinicians gain insight into the disorders and administer more appropriate treatment.

## Clinical manifestations

Due to substantial symptomatic overlap between MD and VM, there exist no highly-specific symptoms that allow for the identification of them. Some core manifestation features may help doctors to make a preliminary judgment when a patient presents for the first time (Table 1).

**Table 1:** Comparisons in clinical characteristics between Meniere’s disease and vestibular migraine

Clinical characteristics	Meniere’s disease	Vestibular migraine
Gender [12, 14, 16–18]		
Female	35~75%	65~86%
Male	37~65%	14~35%
Age of onset (mean value) [12, 14, 16–18, 42]	47.6	42.2
Duration of attacks	20 min to hours	Seconds to days
Headache [12, 14, 16–18, 42, 126]		
Migrainous headache	0~28%	65~76%
Non-migraine headache	6~41%	0~26%
Vertigo [16–18, 126]	90~100%	38~80%
Cochlear symptoms [12, 14, 17, 18, 42, 126]		
Tinnitus	72~98%	0~61%
Aural fullness	37~81%	6~51%
Hearing loss	54~100%	0~79%
Accompany symptoms [12, 14, 17, 18, 42, 126]		
Nausea	20~91%	72~97%
Vomiting	20~74%	50~72%
Photophobia	40~52%	53~86%
Phonophobia	54~63%	10~82%
Previous history of motion sickness [16–18]	20~30%	33~62%
Family history of migraine [12, 16, 17]	16~26%	47~61%

### History and demographic features

Saberi et al*.* [20] concluded that a strong predictor of migraine prevalence in MD was having a positive family history of the condition, since the migrainous MD group registered a higher positive rate of the family history of migraine. The results are consistent with previous findings [21, 22].

Most VM patients reportedly had personal or family history of migraine headaches [23, 24], this finding mimicking that of MD. Nevertheless, a cohort study by van Esch et al. [25] found that patients with VM had a family history of motion sickness, and the highest rate of carsickness episodes, during lifetime, was in pVM and dVM. In fact, motion sickness was endorsed by virtually half of VM patients [23, 24, 26], VM patients are more subject to motion sickness compared with MD, suggesting that there is a hypersensitivity to visual-vestibular mismatch in VM patients.

Additionally, MD tends to develop at an older age and affect males whereas VM usually strikes at a younger age and occurs more in females [17].

The aforementioned findings show that family history and past medical history are two essential features that should not be ignored in the diagnosis of MD and VM. What is more, the family history and past medical history of motion sickness is more related to VM, while people who have a family history of migraine don't necessarily have a definitive diagnosis of VM.

### Headache and migraine-associated symptoms

The main symptom of VM is a headache with or without aura. Various headache symptoms, such as intense pulsating and one-sided headache, might be present in most VM patients, particularly during a typical VM attack, although in nearly 30% of VM patients, vertigo can occur without headache [27]. Vertigo and headache may not always coexist in some persons [28, 29]. It is still debatable if migraine with an aura and vestibular symptoms are related. Some studies suggested such connection [29, 30], whereas other researchers discovered that migraine patients without aura have vestibular symptoms more often or at least at the same frequency as migraineurs with aura [31, 32]. To diagnose VM patients, other migraine features are required, including photophobia, phonophobia [23, 24, 29, 31], and visual aura [23, 29, 33].

Migraine-like symptoms also tend to occur in MD. According to epidemiologic research, the frequency of migraine headaches in MD varies from about 2% to over 80% [21, 34, 35]. In some research, the prevalence of migraine in MD patients was up to twice as high as that in healthy participants (56% vs. 25%, *p* = 0.001), whereas in other investigations, no difference in the rate between these two groups was seen. [12, 34, 36, 37]. Migraine-associated symptoms, including photophobia, phonophobia, and visual auras occurred less often in MD patients, except for some patients with migraine [38].

While there is some overlap in clinical presentations between A and B, more frequent migraine attacks, either during or between episodes, are believed to be typical of VM. It can be claimed that the most sensitive and distinctive characteristic of VM is a history of recurring moderate-to-severe headaches [17]. This proves our case that migraine is a core symptom of VM and can help us distinguish it from MD.

### Vestibular symptoms

Both VM and MD patients may complain of vertigo. However, the duration of vertigo varies. Neff et al*.* [17] found a statistically significant association between hours-long attacks and MD, but, with VM, vertigo could last for a matter of seconds to as long as days. Another study confirms the idea that VM patients' vertigo attacks could last anywhere from a few seconds to many hours, and occasionally even for several days[38].

Wang et al. [39] believe that the monthly frequency of vertigo attacks may be used as a diagnostic marker to distinguish MD patients from individuals who do not suffer from VM. In comparison to MD patients without VM, the average monthly incidence of vertigo attacks among MD patients with VM was noticeably greater (*p* < 0.01).

In VM patients, certain actions like changing positions, head motion or visual motion could generally trigger or aggravate vertigo. The descriptions about vertigo vary greatly, including a sense of spinning, floating, swaying, or feeling off-balance, light-headed [40]. Numerous investigations found that head motion intolerance occurred in 20–85% of VM episodes, positional vertigo in 18–60% of episodes, and spontaneous vertigo in 20–85% of episodes [23, 24, 31, 41]. In MD patients, spontaneous vertigo is the most common chief complaint [3].

In summary, the duration, attack frequency and characteristics of vertigo are major factors that dictate differentiation between MD and VM. With MD, vertigo tends to persist longer, usually for several hours while vertigo in VM has more diverse presentations.

### Aural symptoms: hearing loss, tinnitus and aural fullness

MD patients present a symptomatic pattern dominated by fluctuating hearing loss. Research has concluded that the most sensitive and distinct sign of MD is a history of progressing hearing loss [17]. Lopez-Escamez et al. illustrated that the absence of auditory symptoms, particularly hearing loss, can indicate VM, but the audiometrically found presence of hearing symptoms and hearing loss is indicative of MD [42]. Collectively, the above-mentioned results show that conspicuous and progressive hearing loss, presented either as clinical signs or audiographic abnormalities, is the major symptom of MD rather than VM. Tinnitus and aural fullness are frequent complaints of otological conditions, including both MD and VM, while hearing loss and tinnitus are more typical of common MD [32, 43].

In a nutshell, aural symptoms, especially hearing loss, are found in MD patients more frequently, which can also serve as a distinguishing feature.

### Comorbidities

Though cognitive complaints in patients with VM and MD remain poorly characterized, some relationships between cognitive symptoms and episodic vestibular disorders have been identified. Chari et al. exhibited that in comparison to the MD group, DHI scores were significantly higher in the VM and VMMD groups (DHI score ≥ 31). Cognitive symptoms such as persistent weariness and brain fog, had a relatively high incidence in VM patients [44]. So, we are led to conclude that, compared to MD, VM bore a stronger association with cognitive symptoms.

It is worth noting that VM is more likely to have accompanying/concomitant anxiety, palpitations, and nausea than MD [42]. A study revealed that 50% of VM patients had concurrent psychological conditions, most commonly, anxiety and depression [45]. Although studies have found the rates of psychiatric comorbidities are high in both VM and MD (MD: 57%, VM: 65%) [46, 47], the association between VM and psychiatric disorders (anxiety, depression) was more significant [48].

Since several researches already demonstrated that patients with migraine had higher-than-average incidences of overt depressive and anxiety disorders [49, 50], it is reasonable to speculate that VM patients are particularly susceptible to psychosomatic and mental issues.

## Bedside oculomotor tests

Bedside oculomotor tests are important for the diagnosis of vertigo/dizziness, especially at the first visit. Along with vital symptoms, the signs identified could help clinicians tentatively estimate the origin of disease and further differentiate VM and MD.

### Spontaneous nystagmus

Young et al. [51] found that VM patients characteristically had low velocity ictal spontaneous nystagmus, which could be horizontal, vertical, or torsional. Another study also demonstrated that spontaneous vertical nystagmus was highly specific (93.0%) for VM patients, while spontaneous horizontal nystagmus was specific (82.1%) for MD patients [52]. Both nystagmus of VM and MD can go in different directions, whereas spontaneous vertical nystagmus tenders to suggest the diagnosis of VM since its pathology of VM contains central causes of the condition.

### Head thrust and head-shaking tests

Head thrust and head-shaking tests are widely employed for checking vestibulo-ocular reflex (VOR) function. Head thrust test separately evaluates high-frequency VOR for each ear. It is an important test for the identification of vestibular loss, but it was reportedly insensitive to mild or moderate vestibular loss and unable to detect covert saccades [53]. Currently, this test is increasingly being replaced by the video head-impulse test.

Head-shaking test (HST) is conducted by rotating patient’s head at low frequency (~ 2 Hz) for 20–30 s, and then the head-shaking nystagmus (HSN) was examined. Kim CH et al. [54] found that positive HSN can be seen in 78% of MD patients and 50% of VM patients, the most prevalent kind of HSN in MD was paretic HSN with nystagmus toward the side of the lesion, and downbeat HSN was frequently seen in participants with both MD and VM during follow-up, suggesting that vertical components may be connected to vestibular apparatus involvement and compensation. Another study found that 68% of MD patients had HSN, indicating that HST is a sensitive indicator of peripheral vestibular dysfunction [55].

## Pure tone audiometry

The pure tone audiography is frequently employed in clinical practice as an adjunct in the diagnosis of MD because audiometric testing is standardized and easy to conduct.

McMullen et al. [56] proposed that the duration of disease bore a significant correlation with ipsilateral ear hearing loss (represented by WRS and PTA results), and this may be seen as a hallmark of MD. For MD patients, the mild-to-moderate hearing impairment tends to occur at the early stage of the disease and is followed by a stable period of time after several years [57]. The hearing loss seemed to deteriorate over the duration of the illness [58].

Audiometric abnormalities are unusual in VM, as many studies have shown before. Overall, only a few VM patients had an abnormal hearing at both initial and ensuing tests compared with MD. When hearing loss develops, it can be mild to moderate, unilateral or bilateral and tends to be a low-frequency sensorineural hearing loss that is easily reversible [59, 60]. Patients in both VM and MD groups showed a downward trend over time in pure-tone average (PTA) and low-frequency pure-tone average (LPTA), but in the MD group, the rate of hearing impairment dropped more than in the VM group [59]. Radke A et al.[61] also found that patients with VM often have bilateral symmetric hearing loss, and hearing loss in VM progresses much more slowly, which, up to a point, sets VM apart from MD. Some studies demonstrated that VM patients could develop hearing loss during vertigo attacks [24, 32, 42, 60], but the rate was lower (19–25.7%), especially when compared with MD patients.

By synthesizing previous studies, we see that hearing loss, especially progressive or fluctuating types, are more common in MD patients. So, the worse or unfavorable audiometric results are more indicative of MD. This provides a novel perspective for the differentiation between MD and VM.

## Vestibular tests

Vestibular tests can locate vestibular deficits. It helps, as an adjunct, in the preliminary diagnosis of vestibular disorders and serves to monitor the disease progression and to assess the efficacy during follow-up. Several auxiliary diagnosis tools, such as caloric test (CT), video head impulse test (v-HIT), vestibular autorotation test (VAT), and vestibular-evoked myogenic potentials (VEMPs) can help to detect the dysfunction of the peripheral or central vestibular system and give clues to the diagnosis of vestibular disorders.

### Caloric test

The caloric test (CT) is an objective test that identifies unilateral peripheral vestibular dysfunction but only targets the horizontal semicircular canal. The major indicators of CT include canal paresis (CP), directional preponderance (DP), and reduced vestibular response (caloric asymmetry).

Several studies have yielded normative data. The normal upper limit was 25% for CP, 26% for DP and 22% for caloric asymmetry [62]. To avoid population- or equipment-related variations, each medical center or setting should have its own normative limits.

Prior studies showed that, in 19.6~74.7% of symptomatic MD patients, CT yielded abnormal results. The abnormal results included CP or DP that exceeds the normal upper limits and caloric asymmetry [38, 63–70]. In VM patients, the caloric test could yield abnormal results, which, nonetheless, were limited to the result of CP. The prevalence of abnormal CP in VM subjects stood somewhere from 19 to 42% [38, 64, 67–69, 71, 72], which is much lower than those in MD patients.

To sum up, caloric test could detect abnormalities in both MD and VM patients but more MD patients yielded abnormal results relative to their VM counterparts (Table 2). The results might be ascribed to the following factors. Firstly, caloric irrigation is a non-physiological stimulation in CT, which tests horizontal VOR in the low-frequency range of 0.002–0.004 Hz. Thus, a patient may have a normal result with CT but may still manifest VOR deficits during head movement within higher frequency ranges [73]. Secondly, the pathophysiological mechanism of VM remain unclear and the evidence seemed to support that peripheral and central vestibular deficits both contribute to the pathogenesis of VM [74, 75]. However, the episodic vertigo of VM may simply be a reversible pathophysiological process, involving vestibular pathways, and the caloric test is unable to accurately capture the VOR changes during intermission [72].

**Table 2:** Comparisons in main findings of caloric test between Meniere’s disease and vestibular migraine

Caloric test	Meniere’s Disease	Vestibular Migraine	Authors
Unilateral weakness of caloric response	74.7%	–	Hsun-Mo Wang et al. [63]
	19.6%	0	Martin-Sanz et al. [64]
	65%	–	Stahle et al. [65]
Directional preponderance	38%	–	Stahle et al. [ 65]
	41.7%	–	Sun‑Uk Lee et al. [66]
	6.8%	8%	Mahmut Sinan Yilmaz et al. [67]
Canal paresis	48%	23%	Hye Ran Hong et al. [68]
	47%	25%	Jung Eun Shin et al. [38]
	53.8%	–	Sun‑Uk Lee et al. [66]
	67%	22%	Alexander Blödow et al. [69]
	–	42%	Yoo et al. [71]
	–	19%	Woo Seok Kang et al. [72]
	45.1%	–	Munehisa Fukushima et al. [70]
	66.1%	34%	Mahmut Sinan Yilmaz et al. [67]

Another puzzling issue is that some MD patients yielded normal results with CT. Multiple studies examined the distribution of endolymphatic hydrops (EH) in MD patients and found that the structure of the inferior parts of the inner ear (the saccule and the cochlea) was extensively involved, with the superior sections (the utricle and the semicircular canals) being less affected [76]. In a subset of MD patients, the horizontal semicircular canal might not develop EH, as a result, the CT yielded normal results.

### Video head impulse test

The video head impulse test (v-HIT) is a method that records eye and head velocity and evaluates the gain of vestibular ocular reflex (VOR) of each semicircular canal. Compared with bedside head impulse test, video records can easily detect covert saccades, which may not be captured by human eyes.

This technique is often used as an auxiliary means for the diagnosis of peripheral vestibular diseases. The test parameters include VOR gain, refixation saccades and VOR gain asymmetry (GA).

Some researchers have found that the abnormal rate of v-HIT in MD patients ranged from 27 to 55% [67, 69]. As to VM, Blodow et al.[69]*.* observed only 9% of VM patients with VM had an abnormal gain. The result was consistent with the finding reported by Kang et al. (11%) [72], but substantially lower than the result reported by ElSherif et al*.* (26%) [77]. Martines et al*.* [78] found no significant difference in the horizontal VOR gain between VM and MD patients. Although the results of various studies were not fully consistent, the abnormal rate was generally higher in MD patients than in their VM counterparts (Table 3).

**Table 3:** Comparisons in main findings of v-HIT between Meniere’s disease and vestibular migraine

v-HIT	Meniere’s disease	Vestibular migraine	Authors
Abnormal hVOR gain	–	11%	Woo Seok Kang et al. [72]
	37%	9%	Alexander Blödow et al. [69]
VOR gain asymmetry	–	9%	Yoo et al. [71]
	35.6%	24%	Mahmut Sinan Yilmaz et al. [67]
	–	42.9%	Yollu et al. [127]
Overt saccades	30.5%	8%	Mahmut Sinan Yilmaz et al. [67]
	–	23.8%	Yollu et al. [127]
Covert saccades	6.8%	2%	Mahmut Sinan Yilmaz et al. [67]
	–	33.3%	Yollu et al. [127]

From the aforementioned analysis, we could see that in the majority of VM patients, VOR gain was close to the lower limit of the normal range, and refixation saccades were rare. Due to disrupted inhibitory input from the cerebellar flocculus to the vestibular nuclei, which is a component of the migrainous central spreading depression, the spontaneous neuronal firing rate at the level of these covert saccades can be explained.

Because vestibular tests, when used alone, lack adequate sensitivity in detecting vestibular impairments, it is desirable to use caloric test and v-HIT in combination in the assessment of VOR, as they were employed in many studies. Patients who have normal horizontal v-HIT findings but abnormal caloric test results typically have central lesions, whereas those who have abnormal horizontal v-HIT but normal caloric test results are more likely to have peripheral lesions.

Fukushima et al*.* reported that different caloric test and v-HIT results, in MD patients, might be due to the difference in the severity of the endolymphatic hydrops [70]. Another research by Shugyo et al. [79] supported their assumption. Discrepancies between the caloric test and v-HIT results were observed more in EH patients than in those without EH. A study observed a significantly higher rate of v-HIT caloric dissociation in MD patients [80].

### Vestibular autorotation test

The vestibular autorotation test (VAT) is a commercially accessible computer-based test. The individual is requested to actively move the head at high frequencies (2–6 Hz) during the test to measure both horizontal and vertical VOR responses. This method allows for the measurement of the vestibular ocular reflex over a frequency range similar to the head movement frequency when performing daily tasks.

MD patients typically manifest low horizontal phase and low horizontal gain, as observed by Ng et al. [81]. Hirvonen et al*.* found that the mean gain was lower in MD patients than in controls. When compared to controls, MD patients' phase differences were smaller or slightly shorter, and their asymmetry was greater. What’s more, the MD patients treated conservatively showed a lower abnormality rate than those treated with gentamicin or by surgery [82]. This result might be attributed to the fact that VAT evaluates VOR responses, which may be centrally compensated [83].

A prior study showed that, in VM patients, both horizontal gain (2–5 Hz), vertical gain (2, 4, 5, and 6 Hz) were elevated and horizontal phase was lowered [84]. Another research by Yao et al. yielded similar findings [85], that is, the low horizontal phase plus high horizontal gain helps to exclude MD.

Although the caloric test seems more sensitive than VAT in determining the abnormality of VOR [82], technically it still has some limitations. For example, the result of the test might be affected by the status of neck muscles, such as muscular rigidity, and need the cooperation of the patient. Further research is needed to confirm the reliability of the test.

### Vestibular-evoked myogenic potentials

Vestibular-evoked myogenic potentials (VEMPs) are increasingly used for the diagnosis of vestibulopathy. It includes cervical VEMP (cVEMP) and ocular VEMP (oVEMP), detects dynamic saccular and utricular function respectively.

Compared with oVEMP, cVEMP is more widely used. In cVEMP, clinicians often apply air-conducted (AC) sound stimulus (monaural click or 500 Hz tone burst), and electromyography activity is measured from the ipsilateral sternocleidomastoid muscle before and after stimulation. Valid indicators contain amplitude and asymmetry rate (AR), and normative data between centers are various. In general, when the threshold between 120 and 145 sound pressure level (SPL), the amplitude is between 0.5 and 3 μV, the AR below 35% [86]. In oVEMP, response is measured from infraorbital muscles. Unlike cVEMP, the stimulation used for oVEMP is bone-conduction vibration (BC). The normative data ranges are similar to cVEMP.

#### cVEMP

To date, studies indicated that cVEMP in response to air-conducted sound was abnormal in 40–60% of MD patients, but abnormality rate was lower in patients with VM [51, 87].

Dlugaiczyk et al*.*[88] found that there existed a statistically significant difference in the cVEMP amplitude AR between MD and VM groups. Salviz et al. [89] exhibited that cVEMP could differentiate MD patients (46%) from VM (16%) based on asymmetrically lower amplitudes in the affected ears, with low response rates being at 500 Hz TBs, and altered frequency-dependent responses being at 500 and 1000 Hz TBs in MD patients. Taylor et al. [90]*.* also reported a significantly higher AR for air-conducted sound cVEMP amplitudes in patients with unilateral MD (29%) as compared to VM patients (16%). For MD, the AR of c-VEMP amplitudes increased over time, which was in line with the degree of EH in the affected ear [91]. cVEMP amplitudes in MD patients were more asymmetric than those in VM patients according to these researches, despite some conflicting findings. This suggests that the saccule may be crucial in the pathophysiology of MD.

One of the distinguishing characteristics of cVEMP in MD patients is a shift in preferred frequency [92]. In air-conducted sound cVEMP to stimulation of short tone bursts at 500–700 Hz, healthy people had the biggest amplitudes and the lowest thresholds, whereas MD patients typically showed a shift of a preferred frequency to 1000 Hz [93, 94]. Murofushi et al. [95] proposed the 500–1000 Hz cVEMP slope as an index of the shift in cVEMP tuning properties, *i.e*., an indicator of EH in the saccule. Nonetheless, for VM patients, evidence is lacking that supports the existence of a shift in the frequency of cVEMP. Only one study showed that VM patients experienced a shift of a preferred frequency to 1000 Hz, with latency protracted [94]. The result might be explained by the fact that few VM patients had EH in the otolith organ [96].

The higher abnormal rate of the cVEMP in MD cases may be due to the fact that this test could reflect the status of EH (Table 4). Murofushi et al. [97]*.* found that a positive response in the cVEMP tuning property test corresponded well to EH found by MRI in dMD or pMD patients, this test is useful in detecting EH in MD patients [98, 99]. Since the saccule is the second most common site of hydrops in MD patients [76], we theorize that cVEMP test could be used to evaluate the saccular function and the severity of MD.

**Table 4:** Comparisons in main findings of VEMP between Meniere’s disease and vestibular migraine

VEMP	Meniere’s Disease	Vestibular Migraine	Authors
c-VEMP			
500 Hz air conducted sound	–	11%	Woo Seok Kang et al. [72]
500 Hz bone conducted vibration	64%	39%	Aki Inoue et al. [101]
500 Hz and 1000 Hz air-conducted tone burst	36%	29%	Aki Inoue et al. [101]
o-VEMP			
500 Hz air conducted sound	18%	35%	Aki Inoue et al. [101]
500 Hz bone conducted vibration	25%	21%	Aki Inoue et al. [101]

#### oVEMP

oVEMP abnormalities reportedly accounted for 10–50% in MD patients, but is generally lower in VM patients, standing at approximately 30% (Table 4) [90, 100].

Inoue et al*.* [101] observed the prevalence of AR abnormality with oVEMPs-air (500 Hz tone bursts at 135 dB SPL) was significantly lower in VM cases (57%) than in unilateral MD patients (77%), but no differences were found in the results of o-VEMPs bone, cVEMPs air, or cVEMPs bone, suggesting that the utricular-ocular pathway might be implicated in the pathogenesis of VM.

## Examinations of endolymphatic hydrops

### Glycerin test

Glycerin test, or glycerol test, is believed to temporarily revert the inner ear pressure to normal by reducing the excessive endolymphatic fluid, and thereby improving inner ear function, especially in typical MD patients. Since this effect of glycerol is short-lasting, eventually the hearing threshold remains the same.

A temporary improvement in hearing, due to the effect of glycerol, could be seen in virtually 35.3~58.1% of MD patients [102–104]. Nowadays, clinical data on glycerol use in VM patients are still lacking although some researchers have found EH in some VM patients [96].

Since EH are rarely present in VM patients, and some studies, to date, suggested that EH in MD patients are more severe and common, the association between glycerin test and the core symptoms may serve to distinguish between MD and VM.

### Electrocochleography

Electrocochleography (EcochG) has been used to test the functional status of the peripheral auditory tract. It counts the cochlear potentials that sound stimulation elicits. The summating potential (SP) and the action potential (AP) obtained from EcochG are used to calculate the SP/AP ratio in percent. In general, an SP/AP ratio above 40% is taken as abnormal [105].

Quatre et al. [106] reported that abnormal SP/AP ratios of EcochG were found in 68% of definite MD patients. In another clinical study, Francesco Martines et al. [78] found a higher proportion of abnormal SP/AP ratio in MD patients (52.38%) than in their VM counterparts (14.28%). These results suggest that EcochG is helpful in the diagnosis of MD, a higher SP/AP may result from EH-caused displacement of basilar membrane.

Pou et al*.*[107] reported that the sensitivity of ECochG for the diagnosis of EH was 57%. A study by Kim et al. [108] yielded a similar sensitivity (66.7%). Therefore, the abnormally higher SP/AP ratio is highly suggestive of EH, which same as the specificity of this test. It has been found to have a false-positivity result in few special cases, such as perilymphatic fistula [109]or superior canal dehiscence syndrome [110]. In other words, since MD has been identified as an idiopathic condition of EH, ECochG may be an essential component of the diagnostic test battery (Table 5) [111].

**Table 5:** Comparisons in main findings of electrocochleography between Meniere’s disease and vestibular migraine

Electrocochleography	Meniere’s disease	Vestibular migraine	Authors
SP/AP ≥ 0.4	66.7%	–	Harold et al. [108]
SP/AP ≥ 0.43	68%	–	Raphaële et al. [106]
SP/AP ≥ 0.5	35.3% –	10% 38.1%	Martin-Sanz et al. [64] Yollu et al. [127]
Tone burst EcoG Clicks EcoG	83% 30%	– –	Jeremy et al. [105] Jeremy et al. [105]

### Inner ear imaging

Imaging technologies are the pillar for in vivo assessment of endolymphatic and perilymphatic structures in both MD and VM patients.

Some experts believe that to establish a reliable diagnosis of MD, histopathological proof of EH is required [112]. Gadolinium-contrasted magnetic resonance imaging (Gd-MRI) now has allowed us to visualize EH in human in vivo.

For the discrimination between MD and VM, Nakada et al. [96] suggested endolymphatic space size differed between VM patients and vestibular MD patients with headache. The size was morphologically assessed by 3 T Gd-MRI. Patients with VM (21%) seldom showed EH in vestibule, while vestibular EH was more often found in MD patients (100%). Sun et al. [113], by employing Gd-MRI, found that the affected ear of MD patients had EH of various degrees in their cochlea and vestibule (100%), but only suspicious cochlear hydrops or no vestibular hydrops was noted in the VM patients (10%). Gürkov et al. [114]*.* demonstrated that few VM patients with auditory symptoms presented EH on Gd-MRI, and other scholars demonstrated that EH was present in all definite MD patients [115].

On the basis of the aforementioned discussion, we are led to conclude that that the frequency and location of EH are different between VM and MD. In MD patients, both vestibular and cochlear EH are frequently detected by Gd-MRI, whereas EH is rare and principally restricted to cochlea in their VM counterparts. Inner ear imaging, especially Gd-MRI, may be a powerful tool in the differentiation between VM and MD.

## Biomarkers

A great many scientists are endeavoring to identify specific serum biomarkers for the differentiation between vestibular diseases, such as MD and VM. Unfortunately, no significant results have been achieved so far.

Naples et al. [116] demonstrated that pristin and stolen-1 were all elevated in MD patients. But they are ineffective in differentiating MD from VM.

Since a theory holds that the immune system plays a crucial role in a large population of MD patients, there is more study being done on biomarkers of MD [111]. Flook et al. [117] found MD and VM patients had different pro-inflammatory signatures, a cytokine panel, including IL-1β, and CXC-subfamily chemokines (CCL3, CCL22, CXCL1). They observed that IL-1β, and CCL3 were elevated in MD patients, while in VM patients, CCL22 and CXCL1 were higher. Moleon et al. [118] described that the levels of CCL18, CCL3, and CXCL4 differed between patients with MD or migraine and controls, but no difference was found in the level of IL-1β, which is inconsistent with the aforementioned results. Even while there is evidence to support the idea that immune variables may play a role in the onset of MD, there are currently no reliable immunological biomarkers available for the diagnosis of MD or VM.

## Longitudinal follow-up

Due to the heterogeneity of the manifestation and difficulty in differentiation, the initial diagnosis may be revised or the diagnosis remains tentative or uncertain. A study found striking relative increases of final diagnoses of VM relative to referral diagnoses in patients < 65 years (1.8–20.2%) [119]. Diagnosis of MD remains a challenge due to the absence of typical or specific presentations, especially at the early stages. A study showed that 50.43% of MD patients (*n* = 58), initially presented only cochlear symptoms without vertigo [120]. Besides, Andrea Radtke et al. reported that, after a median follow-up time of 9 years, seven patients with VM had cochlear symptoms and hearing loss that formally satisfied AAO criteria for bilateral MD, and clinical features were atypical of MD [61].

Even against the current diagnostic guidelines of VM and MD, a sizable number of individuals with sudden or sporadic vestibular symptoms cannot be definitively diagnosed. Dlugaiczyk et al. [121] defined this heterogenous group of patients as “recurrent vestibular symptoms not otherwise specified” (RVS-NOS). This condition has a stable symptom profile over time, and it doesn’t possess features specific enough to be a disease entity. Researchers thus came to the conclusion that RVS-NOS is more likely to comprise a range of moderate or insufficient/incomplete forms of well-known vestibular diseases, like VM and MD. van Leeuwen et al. [122] agreed with this aforementioned notion, and believed that benign recurrent vertigo (BRV) is a subgroup of RVS-NOS. They are highly comparable to VM than MD, especially in the width of the spectrum of vestibular symptoms, and frequency of vertigo attacks. Although a majority of previous research revealed BRV patients had a positive course with a significant decrease in attack frequency, a few cases still progressed into VM [123].

Therefore, it is necessary to dynamically observe the disease progression and response to initial therapy, in order to establish a definitive diagnosis or revise our initial diagnosis. This revised diagnosis might lead to more appropriate and effective therapy.

## Differentiation of MD and VM: diagnostic battery

Up till now, the exact mechanism of VM and MD is not fully elucidated. The generally accepted theories on VM mainly include: (1) the activation and sensitization of the trigeminal vascular system cause the vasospasm in internal auditory and brain arteries; (2) brain ion channels disrupted; (3) cortical spreading depression theory [124]. With MD, EH is believed to be the main histopathological lesion of MD, and may mechanistically result from other pathophysiological events. In conclusion, the central nerve system and the vascular system both play important roles in the development of VM, whereas MD is assumed to be a peripheral vestibular disorder.

**Fig. 1:**
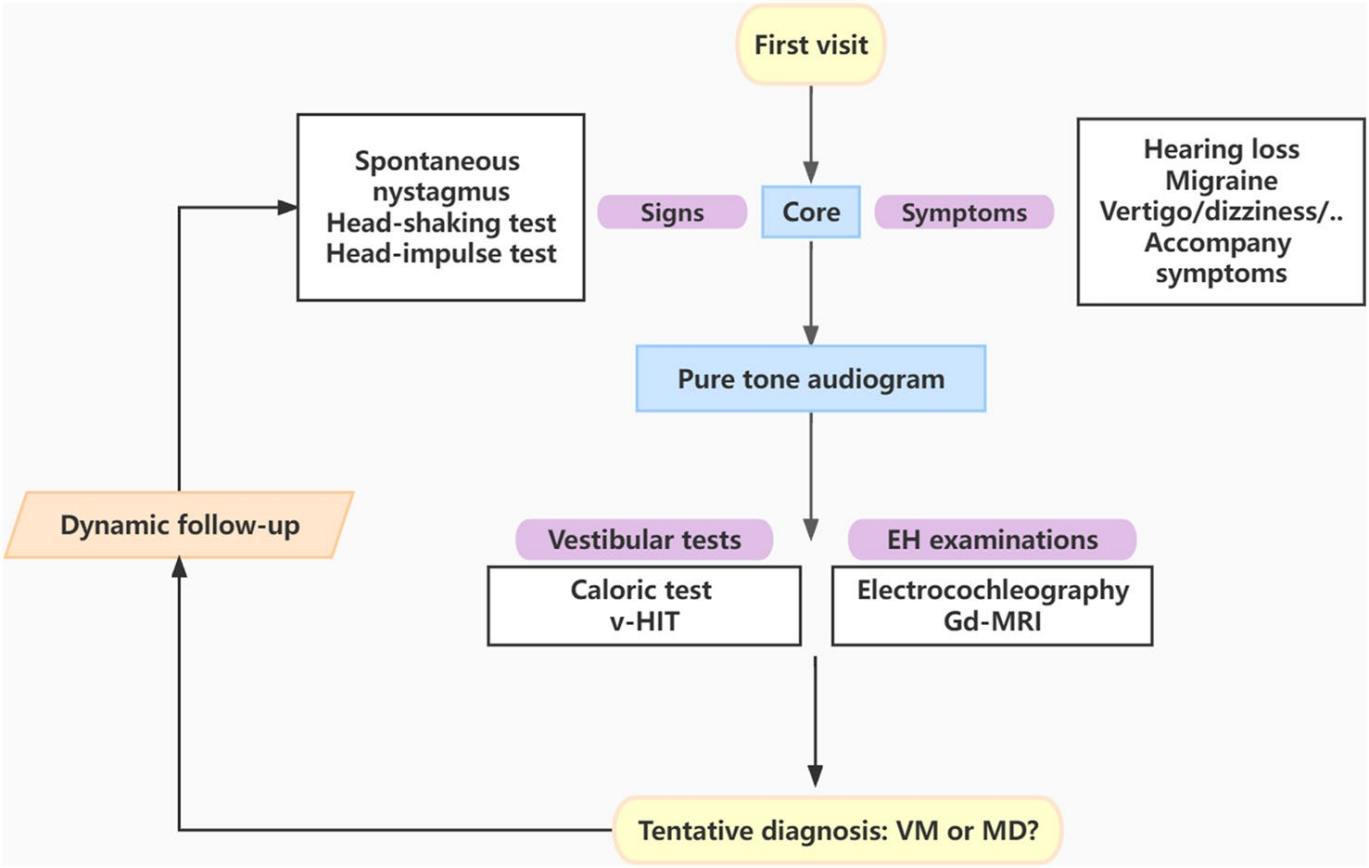
The differenetiate diagnosis of suspicious process of vestibular migraine (VM) and Meniere’s disease (MD) patients

Since we have demonstrated overlaps in many aspects of these two diseases, which means no single test is specific enough on its own. We assume that the patterns including some core diagnostic elements would increase the specificity of differentiation. This diagnostic battery should cover core clinical symptoms and signs, and include related auxiliary tests (audiometry, vestibular tests, EH examinations). With the use of the battery, a careful follow-up is also required for definitive differentiation between the two conditions (Fig. 1).

Diagnostic battery begins with the test of core clinical presentations. For VM patients, vertigo attack is more frequent and lasted variously from seconds to several days. Headache is common and accompanied by autonomic symptoms, photophobia, phonophobia or other typical migraine features. Some patients may have hearing loss, which, nonetheless, hardly fluctuates or deteriorates. With MD patients, vertigo attacks are less frequent, and persist no more than hours. In practically all MD patients, hearing loss that fluctuates and deteriorates is a major symptom. Tinnitus and aural fullness are constantly seen in MD patients but are much less in VM patients.

Next comes PTA, a basal and essential test. PTA, in MD patients, conspicuously shows hearing impairment at low frequency, and follow-up reveals symptom fluctuation and frequency expansion to all frequencies. Nevertheless, the PTA abnormality is unusual in VM patients, a few VM patients suffer from mild-to-moderate hearing loss at high frequency.

The vestibular function tests plus EH-detection may better inform the differentiation between VM and MD. Although EH is not definitively indicative of the diagnosis of MD, it is still helpful in the discrimination between MD and VM, since VM patients seldom exhibit EH. In regard to vestibular tests, CT in combination with v-HIT could cover all frequencies of VOR response, and abnormal results indicate peripheral vestibular hypofunction, which is more associated with MD. As for the detection of EH, Gd-MRI may be a good choice since EH can be visualized with high sensitivity and specificity. In some medical centers with limited resources, EcochG can be a good substitute for Gd-MRI.

For early diagnosis of MD and VM, differentiation of the two diseases still poses a great challenge. Some MD patients in the early stage may manifest recurrent vertigo with tinnitus or ear fullness as main complaint, and as we elucidated before, VM patients would show vertigo and tinnitus as well, so initial clinical symptoms of MD and VM are comparable. Given the possibility of misdiagnosis at the early stage, auxiliary examinations, as important complements, are indispensable. Audiographic results should be obtained in any suspected patients, on the target to identify hearing loss without subjective sensation. Fukushima et al. [125]*.* demonstrated that, at the early stage of MD, deterioration in the vertical semicircular canals tends to go before exacerbation in the horizontal semicircular canal, so it is reasonable to theorize that v-HIT could be a useful tool for differentiation at the early stage. What is more, the EH examinations (EcochG, Gd-MRI) can yield more telling findings at early stages, consindering that EH plays a pivotal role in the pathogenesis of MD.

## Conclusions

In view of the evident overlap in clinical symptoms and intrinsic pathogenesis, more attention should be paid to the differentiation between MD and VM. It is high time we gave up the mindset that over-emphasizes clinical signs of vestibular diseases and adopted a multiple-perspective approach that focus on the core symptoms and signs, audiologic function, vestibular function, and follow-up. This multiple-pronged approach entails that, at the initial diagnosis stage, vestibular-auditory tests should weigh as much as history taking, to avoid mis-diagnosis up to the hilt. In the follow-up, the clinical course of the disease and the efficacy of the initial therapy should be meticulously monitored to confirm or revise our initial diagnosis.
